# Data from long time testing of 18650 lithium polymer batteries

**DOI:** 10.1016/j.dib.2020.105137

**Published:** 2020-01-15

**Authors:** Martin Novak, Jan Chysky, Lukas Novak

**Affiliations:** Department of Instrumentation and Control Engineering, Faculty of Mechanical Engineering, Czech Technical University in Prague, Technická 4, 166 00, Prague, Czech Republic

**Keywords:** Battery testing, 18650 batteries, Battery charging/discharging, Battery ageing, Battery capacity vs temperature

## Abstract

This is a data set from long time testing of 18650 Lithium polymer batteries. It includes the following batteries: Samsung ICR18650-30B (2950 mAh), Joy NCR18650A300A (3000 mAh), GP 03624RC 3.6 V (3000 mAh) and EVC 200L06C (2000 mAh). For measurement, four batteries are connected in parallel and are charged and discharged with a controlled current of 1 A per battery. The current is controlled with a NI cRIO control system programmed in LabView. Battery current is measured with LEM LA 55-P current transducers; voltage is measured directly with the cRIO's analog inputs. The data set contains measurement for between 1200 and 1940 full charging/discharging cycles. The data set can be reused to get information about the dependence of battery capacity vs. number of cycles. Temperature of the battery is measured, therefore the dependence of capacity on temperature can be found out as well.

**Table:** Specifications Table

Subject	Electrical and Electronic Engineering
Specific subject area	Properties of Lithium polymer batteries
Type of data	Tables
How data were acquired	Control system cRIO-9073 with analog input module NI 9215 and digital output module NI9472, National Instruments, Austin, TX, USA, software LabView version R2014R1 LEM LA 55-P current transducers, LEM International SA, Plan les Ouates, Switzerland Power supply Manson HCS-3602-000G, Kwai Chung, N.T., Hong Kong
Data format	Raw data of battery voltage, current, temperature and number of current charging/discharging cycle Sampling time is 3min
Parameters for data collection	The batteries were charged/discharged with constant current. The current was controlled with PID algorithm implemented on the cRIO system.
Description of data collection	The cRIO system measured the battery voltage and controlled the current. Based on voltage value, the battery was charged/discharged. The battery was charged until voltage of 4.15 V was reached and discharged to 3.0 V. Temperature of the batteries was measured with a temperature sensor KTY11 placed under the battery.
Data source location	Institution: Czech Technical University in Prague, Faculty of Mechanical Engineering, Department of Instrumentation and Control Engineering City: Prague Country: Czech Republic
Data accessibility	Electronic data set is with this article, as a set of one.zip file

**Table:** 

**Value of the Data** • This data set provides publicly available data for long time battery testing. It may be used in estimation of battery lifecycle and degradation models, e.g. Ref. [1], or to create models for state of charge estimation for batteries using a model based approach, e.g. Ref. [3]. • The data set can be used by researchers. Researchers working on the design of electric vehicles can use the data to create battery models with estimate of the battery lifetime cycle. • The data can be used to develop more advanced models for lithium batteries lifecycle prediction and parameter uncertainties, e.g. Ref. [2], including verification with experimental data. • Educators can use the dataset for teaching and instructional purposes. • The dataset can be used to study the dependence of battery capacity on temperature in order to select less temperature sensitive batteries where this is required by the application. • The dataset can be used by electric car manufacturers to make models for prediction of manufacturing and lifecycle costs of battery electric vehicles, e.g. Ref. [4].

This data set is new and original, and the data has not been published elsewhere.

## 1 Data description

The battery parameters and data set filenames are shown in Table 1. The table lists the setup #, battery model and manufacturer, nominal capacity, charging and discharge cut-off voltage. All batteries were size 18650 cells.

**Table 1:** Battery parameters and data set filenames.

Setup #	1	2	3	7
Battery model	ICR18650-30B	NCR18650A300A	GP 03624RC 3,6 V	200L06C
Manufacturer	Samsung	Joy	GP	EVC
Nominal capacity (mAh)	2950	3000	3000	2000
Charging voltage (V)	4.35 V ± 0.05	4.2 ± 0.03	4.2 ± 0.03	Not available
Discharge cut-off voltage (V)	2.75	2.5	Not specified in datasheet, given as “standard discharging method 1C constant current discharge to 2.75V″	Not available
Datasheet reference	[5]	[6]	Not publicly available	Not available
File name	bat #1.txt	bat #2.txt	bat #3.txt	bat #7.txt

The data file structure is shown in Table 2. The sampling time is 3min.

**Table 2:** Data file structure.

**Column 1** – date, format yyyy-MM-dd
**Column 2** – time, format HH:mm:ss
**Column 3** - Battery current (A). Positive current = charging, negative current = discharging. 4 batteries are connected in parallel, so individual current is (column 3)/4. The individual battery current is not measured.
**Column 4** – Battery voltage (V)
**Column 5** – Battery pack temperature (°C), measured with a temperature sensor KTY11 placed under the battery
**Column 6** – number of current charging/discharging cycles from the beginning of the experiment for corresponding setup #. When some data (cycles) are missing, the data is not available due to data loss

The raw data was used to calculate the battery capacity that was discharged during the discharging cycle. The discharged capacity was calculated as a time integral of current over the length of the discharging cycle. The average battery temperature is the calculated average temperature during the battery discharging cycle and is calculated from the instantaneous temperature in column 5.

The supplied code was created in Matlab2017a.

**Fig. 1:**
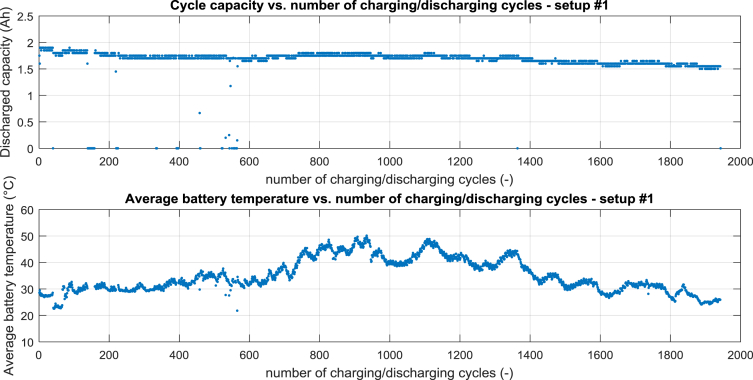
Processed data for setup #1, top cycle capacity vs. number of charging/discharging cycle, bottom average battery temperature vs. number of charging/discharging cycles.

For setup #1 the cycle capacity vs. number of charging/discharging cycles is shown in Fig. 1 – top, the average battery temperature vs. number of charging/discharging cycles is shown in Fig. 1 – bottom.

**Fig. 2:**
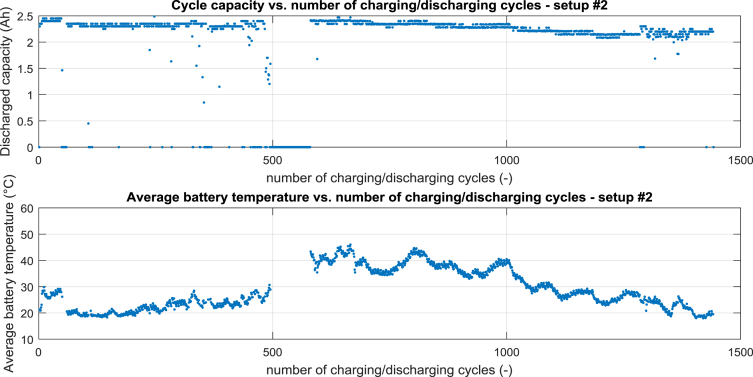
Processed data for setup #2, top cycle capacity vs. number of charging/discharging cycle, bottom average battery temperature vs. number of charging/discharging cycles.

For setup #2 the cycle capacity vs. number of charging/discharging cycles is shown in Fig. 2 – top, the average battery temperature vs. number of charging/discharging cycles is shown in Fig. 2 – bottom.

**Fig. 3:**
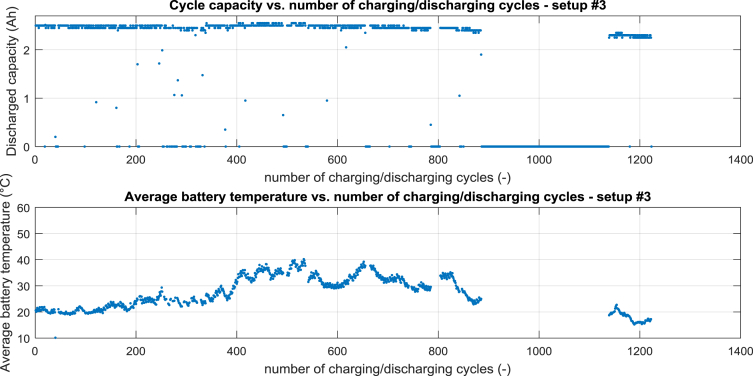
Processed data for setup #3, top cycle capacity vs. number of charging/discharging cycle, bottom average battery temperature vs. number of charging/discharging cycles.

For setup #3 the cycle capacity vs. number of charging/discharging cycles is shown in Fig. 3 – top, the average battery temperature vs. number of charging/discharging cycles is shown in Fig. 3 – bottom.

**Fig. 4:**
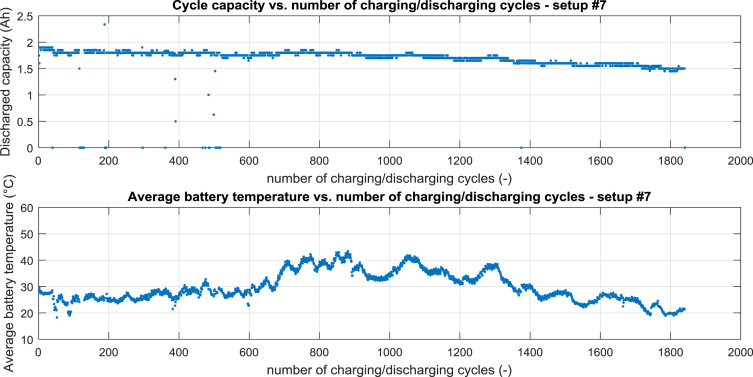
Processed data for setup #7, top cycle capacity vs. number of charging/discharging cycle, bottom average battery temperature vs. number of charging/discharging cycles.

For setup #7 the cycle capacity vs. number of charging/discharging cycles is shown in Fig. 4 – top, the average battery temperature vs. number of charging/discharging cycles is shown in Fig. 4 – bottom.

## 2 Experimental design, materials, and methods

**Fig. 5:**
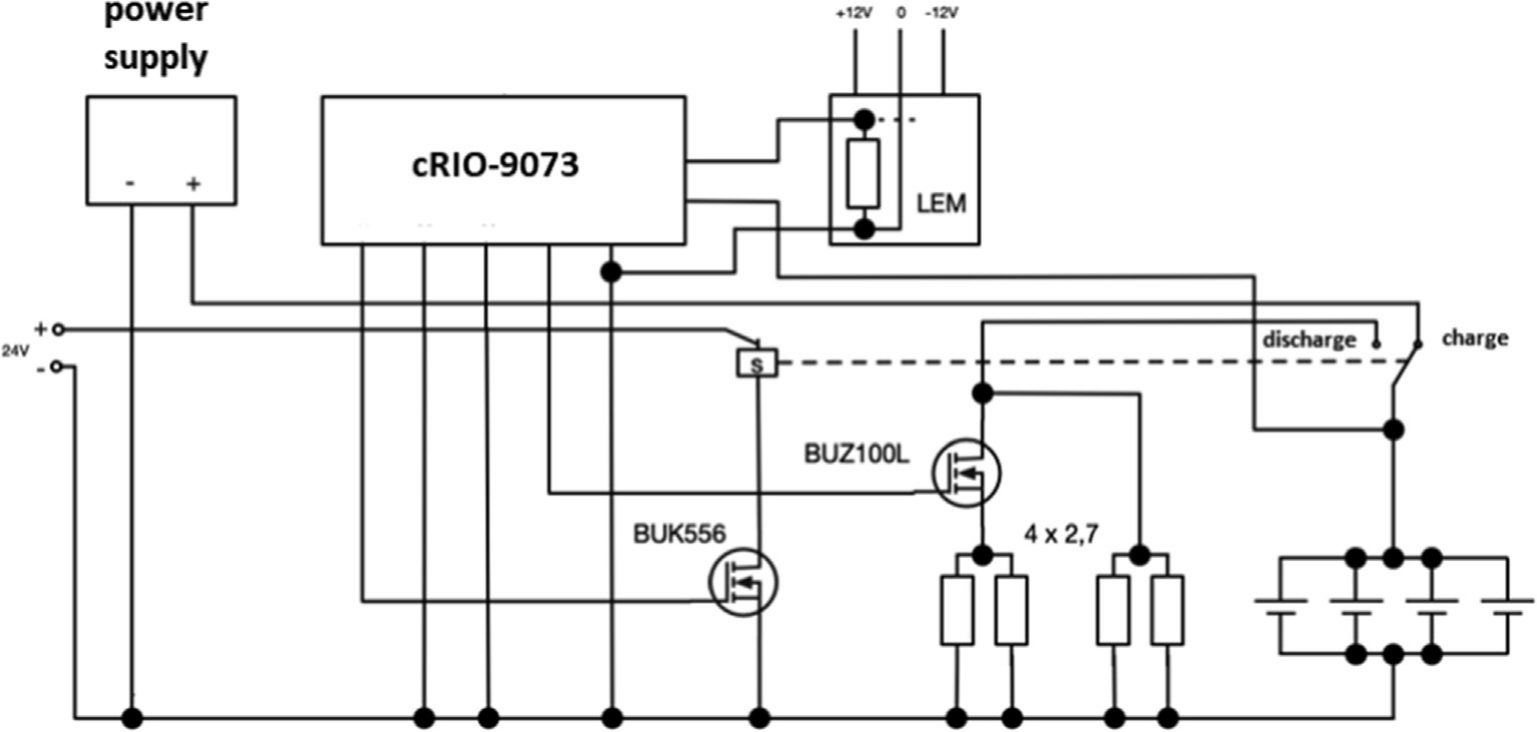
Block diagram of the charging/discharging experiment.

The block diagram of the charging/discharging experiment is shown in Fig. 5.

Control system cRIO-9073 with analog input module NI 9215 and digital output module NI9472, software LabView version 12, LEM LA 55-P current transducers, Power supply Manson HCS-3602-000G. cRIO system measured the battery voltage and controlled the current. The current is measured with the LEM current sensor. Based on voltage value, the battery was charged/discharged.

The charging is stopped when the cell voltage is equal to 4.15V. Then the discharging cycle begins immediately. The discharging is stopped when the cell voltage is smaller or equal to 3.00 V, then the charging cycle starts immediately. Those values were specified by an electric bus manufacturer who first initiated this long time measurement.

The charging and cut-off voltages in Table 1 are taken from the referenced datasheets, when the datasheets are available or public. For setup #3 the datasheet is confidential, for setup #7 the datasheet is not available at all.

Temperature of the batteries was measured with a temperature sensor KTY11 placed under the battery.

The testing is done in a university lab. Without temperature control in the room. The testing is not done in an environmental chamber. Therefore the temperature is varying between summer and winter. Our estimate is between 30 °C in summer on hot summer days and 15 °C in winter. The ambient temperature is not measured. The effect of fluctuations of the ambient temperature is attenuated (not entirely) by the experimental setup. The battery cells are all enclosed in metal PC cases, dimensions 360 × 395 × 95 mm. The heat from charging and discharging the batteries heats the inside of the case and attenuates partly the fluctuations of the temperature in the room.

The acquired data were not filtered.

**Fig. 6:**
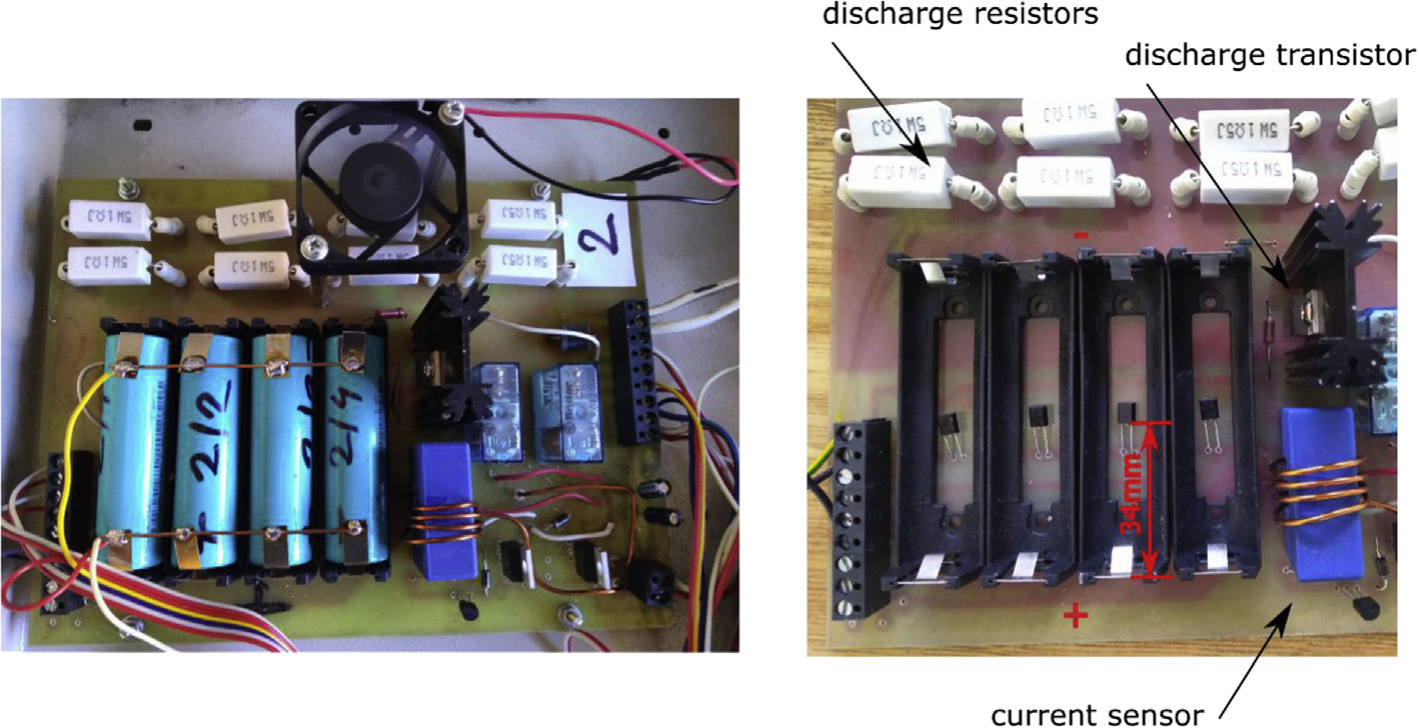
Left - Experimental setup, the welded tabs are used to measure the battery voltage, right – detail of the temperature sensor placement, batteries removed from battery holders.

The placement of temperature sensors under the battery is shown in Fig. 6 right. The sensor is aligned at a distance of 34mm from the external edge of the + terminal of the battery holder placing it approximately in the center of the battery. The battery length is 65 mm excluding the holder, including the holder it is 75 mm. The temperature sensor is pressed against the battery, there is no heat conductive compound between the sensor and battery.

In each setup the temperature is measured for each battery, so each setup has 4 battery temperature sensors. The temperature in the provided files is the average temperature of all 4 batteries in a single setup.

In order to prevent the measurement of voltage drop between the battery and the battery holder, the batteries have welded tabs. The voltage is measured directly on those tabs to prevent the influence of contact resistance at the holder/battery transition. The measurement setup is shown in Fig. 6 right.

## 3 Expected experimental uncertainty

The current is measured with an LEM LA 55-P current transducer, specified accuracy ±0.90%; the signal is then sampled with the NI 9215 analog input module, the same as for the voltage input.

The voltage is measured directly with the analog input of the control system cRIO-9073 with analog input module NI 9215, specified accuracy ±0.60% of reading (Uncalibrated, typ (25 °C, ±5 °C)), resolution 16 bits in range ±10V.

Temperature is measure with a KTY11 Silicon Temperature Sensor, specified accuracy ±3%
